# Using Metabolic Engineering to Connect Molecular Biology Techniques to Societal Challenges

**DOI:** 10.3389/fmicb.2020.577004

**Published:** 2020-11-16

**Authors:** Claire L. Gordy, Carlos C. Goller

**Affiliations:** ^1^Department of Biological Sciences, North Carolina State University, Raleigh, NC, United States; ^2^Biotechnology Teaching Program, North Carolina State University, Raleigh, NC, United States

**Keywords:** metabolic engineering, yeast, sustainability, case study, molecular biology, group work, agency, ownership

## Abstract

Genetically modified organisms (GMOs) are a topic of broad interest and are discussed in classes ranging from introductory biology to bioethics to more advanced methods-focused molecular biology courses. In most cases, GMOs are discussed in the context of introducing a single protein-coding gene to produce a single desired trait in a crop. For example, a commercially available kit allows students to test whether food products contain GMOs by detecting the *Bacillus thuringiensis* delta-endotoxin gene, which confers resistance to European corn borers. We have developed an 8-week laboratory module for upper-division undergraduates and graduate students that builds upon students’ basic understanding of GMOs to introduce them to the techniques used to sustainably produce commercially valuable products in yeast through metabolic engineering. In this course, students use recombination-based methods to assemble genes encoding entire metabolic pathways in *Saccharomyces cerevisiae*, perform genetic screens to identify yeast genes that impact metabolite yield, and use error-prone PCR to optimize metabolic pathway function. In parallel to these laboratory-based activities, students engage with the societal impact of these approaches through case studies of products made via yeast metabolic engineering, such as opioids, omega-3 fatty acids, and the Impossible Burger. In this report, we focus on these case studies as well as an individual sustainability project assignment created for this course. This assignment, which spans the 8-week module, asks students to find examples of yeast metabolic engineering that could be used to address current sustainability challenges in their communities. By the end of the course, students synthesize this information to create a case study that could be used to teach concepts related to metabolic engineering and sustainability to their peers. Student approaches to this project have varied from literature reviews, to news searches, to directly contacting and interviewing researchers using novel metabolic engineering approaches. These student-produced projects are used as case studies in future semesters, amplifying student voices and contributing to student ownership. While developed in the context of this course, the sustainability project and case studies are broadly applicable and could be adapted for use in biology or bioethics courses at the undergraduate or graduate level. Through this report, we hope to gain collaborators interested in implementing a version of the course at their institutions, allowing for robust assessment of the impact of the course on a larger group of students.

## Introduction

As arguably one of the best-characterized, safest, least expensive, and most genetically tractable model organisms, *Saccharomyces cerevisiae* is an appealing choice for use in teaching labs (Sitaraman, 2011; Wolyniak, 2013; Bowling et al., 2016; Oelkers, 2017; Hageman and Krikken, 2018; Pedwell et al., 2018; Sehgal et al., 2018; de Waal et al., 2019). Yeast has been used as a model system for a variety of different courses and educational activities. Students have worked with *S. cerevisiae* to learn about Mendelian genetics and molecular biology concepts (Wolyniak, 2013), deletion of genes in yeast (Hageman and Krikken, 2018), transcriptional regulation (Oelkers, 2017), and the creation of frameshifts using CRISPR/Cas9 (de Waal et al., 2019). Several published course-based undergraduate research experiences (CUREs) allow students to contribute novel findings while learning fundamental molecular biology and genetics skills (Bowling et al., 2016; Pedwell et al., 2018). Wild yeasts have also been used to engage the public as citizen scientists with at-home experiments (Nichols, n.d.). The availability of curated databases (e.g., Saccharomyces Genome Database SGD) (Saccharomyces Genome Database [SGD], n.d a), genetic tools, and adaptable protocols make *S. cerevisiae* an attractive and powerful model system for lab-based courses that can transform our students as scientists.

In redesigning an existing yeast genetics course, our dual goals were to implement the elements of a course-based research experience (CRE) and to engage students in thinking beyond the science to its social implications. As we began this redesign, we were inspired by Jef Boeke’s lab’s work developing synthetic biology tools in yeast. In the redesigned course, we aimed to use the Yeast Golden Gate (yGG) and the versatile genetic assembly system (VEGAS) methods created by the Boeke lab (Agmon et al., 2015; Mitchell et al., 2015) to engage students in metabolic engineering of yeast to produce the red-orange vitamin A precursor beta-carotene, which is used in foods, feeds, cosmetics, and pharmaceuticals (Nelis and Leenheer, 1991; Anunciato and da Racho Filho, 2012; Li et al., 2013). The lab component of the course emphasized applications of yeast metabolic engineering, while the lecture focused on sustainability and the societal implications of the use of engineered yeast as “sustainable” sources of commercial products. While a robust evaluation of the impact of the course redesign on student engagement will require assessment of multiple cohorts of students over several years, student participation in class discussions, informal conversations during lab sessions, and enthusiasm during end-of-semester poster presentations suggest that the CRE structure facilitated engagement.

## Course Description

**FIGURE 1:**
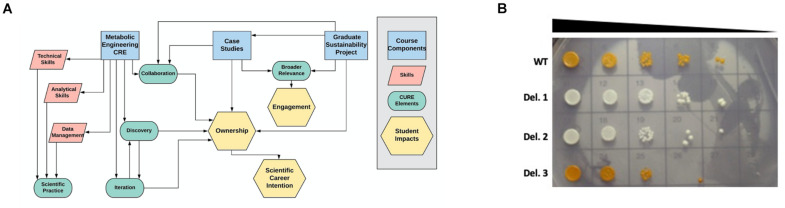
Course design and example student data. **(A)** The three main components of the Yeast Metabolic Engineering course (blue rectangles) were designed to train students in specific skills (pink parallelograms) while incorporating the five elements of course-based undergraduate research experiences (green ovals), leading to the student impacts of engagement, ownership, and scientific career intention (yellow hexagons). **(B)** Students performed VEGAS to assemble the beta-carotene biosynthetic pathway in wild type (WT) yeast or a pool of barcoded single gene deletion strains. Transformants displaying decreased beta-carotene production (white colonies) or increased beta-carotene production (dark orange colonies) were selected for spot plate analysis to compare genetic fitness and barcode sequencing to identify the deleted genes. In some cases, these experiments resulted in novel findings. Deletion strain 3 (Del. 3) in this student experiment displayed increased accumulation of beta-carotene, and barcode sequencing identified this strain as an Mcr1 deletion mutant. This finding suggests that interfering with competing pathways, such as the ergosterol biosynthetic pathway, can drive increased beta-carotene accumulation.

The Yeast Metabolic Engineering course is an 8-week long lab module enrolling juniors, seniors, and graduate students from across multiple majors, programs, and colleges at NC State University in Raleigh, NC. It is structured as one 2-h lecture session and one 5-h lab session per week and housed within the Biotechnology Teaching Program (BIT). As a BIT course, it is designed to teach students cutting-edge technical skills that can be applied in academic research or in industry. The prerequisite for enrolling in lab modules is the completion of a semester-long lab-based molecular biology course (Carson et al., 2019; Garcia et al., 2020). As technology changes, BIT courses are regularly redesigned and updated to reflect current research and molecular biotechnology tools. As we worked to redesign this course, our two goals were: (1) Adopting elements of a course-based research experience (CRE) in the lab, and (2) Engaging students in thoughtful discussion of the societal implications of yeast metabolic engineering, with a focus on sustainability (Figure 1A).

The course was developed using backward design, with the following course goals:

1. **Design** and **troubleshoot** experiments to grow and genetically manipulate *S. cerevisiae*.
  a. **Verify** the genotype of yeast strains.
  b. **Engineer** constructs for overproduction of beta-carotene.
  c. **Transform** yeast with recombinant DNA constructs.
  d. **Assess** the viability of genetically engineered yeast.
  e. **Measure** the intensity of pigmentation (beta-carotene) in different strains.
2. **Interpret** data associated with metabolic engineering of yeast.
3. **Identify** limitations and alternative approaches associated with the metabolic engineering of yeast.

Weekly lecture and lab sessions had assignments with objectives designed to align with these course goals. Assessments included quizzes, case studies, reading assignments, electronic lab notebook entries, lab reports, posters, a final exam, and a sustainability project for graduate students.

## Lab Component: Scientific Practice, Collaboration, Discovery, and Iteration

Of the 8 weeks in this course, students spend 6 weeks working to optimize the production of a commercially useful metabolite in *S. cerevisiae*. In the current iteration of this course, students produce beta-carotene, a vitamin A precursor. This particular product was selected because students can easily perform initial phenotypic screens, as beta-carotene-producing colonies are visibly orange. Production of beta-carotene requires the introduction of three exogenous genes encoding enzymes that catalyze the stepwise conversion of acetyl coA to beta-carotene, and optimal production is obtained when the gene encoding a truncated form of Hmg1, an endogenous *S. cerevisiae* protein, is overexpressed (Verwaal et al., 2007; Li et al., 2013; Mitchell et al., 2015). To achieve this, students assemble these four genes, along with a KanR selection cassette, using the versatile genetic assembly system [VEGAS; (Mitchell et al., 2015)]. VEGAS exploits the capacity of *S. cerevisiae* to join sequences with terminal homology by homologous recombination: students transform yeast with a digested acceptor plasmid and individual transcriptional units (TUs) consisting of a promoter, gene of interest, and terminator, each flanked with orthogonal adapter sequences (Agmon et al., 2015). These adapter sequences are designed with terminal homology such that homologous recombination assembles the genes in the desired order into the plasmid (Mitchell et al., 2015).

To increase student engagement and ownership of the lab-based project, we have incorporated four elements of course-based research experiences supported by evidence as best practices: scientific practice, collaboration, discovery, and iteration (Auchincloss et al., 2014; Brownell and Kloser, 2015; Corwin et al., 2018; Figure 1A). Importantly, collaboration, discovery, and iteration have been shown to increase not only student ownership but also students’ intention to pursue a scientific career (Corwin et al., 2018).

Collaboration is achieved through both the laboratory and lecture portions of the course. In the lab, students work in pairs to carry out research projects that contribute to the overarching goal of identifying genetic changes that improve beta-carotene yield. Thus, each student collaborates with their partner, while the pairs collaborate as one larger research group. In the lecture portion of the class, students can choose to work in groups on in-class activities, case studies, and the graduate sustainability project. The groups students form for these activities and assignments tend to include 3–4 students, allowing for collaboration within larger groups than in the lab. In contrast to most CUREs, our course enrolls both undergraduates and graduate students. This allows for collaboration among students at different academic levels and the formation of “near-peer” mentoring relationships, which have been shown to benefit both the mentor and the mentee (Tenenbaum et al., 2014; Trujillo et al., 2015).

While the lab portion of the class utilizes published techniques for assembling a well-characterized metabolic pathway, the element of discovery is added by performing two different genetic screens to further optimize metabolite production. This approach differs from other courses in which students produce beta-carotene in yeast, such as the Cold Spring Harbor Laboratory Yeast Genetics and Genomics course (Dunham et al., 2015), which focus on techniques rather than experimental design and inquiry.

Our students engage in discovery through two different genetic screening experiments: (1) Assembly of the beta-carotene pathway in a pool of barcoded yeast gene deletion strains (Winzeler et al., 1999; Giaever et al., 2002), followed by barcode sequencing of beta-carotene over- or under-producers to identify novel genes involved in beta-carotene production; and (2) Error-prone PCR amplification of TUs to optimize beta-carotene production through random mutagenesis of the genes encoding the metabolic pathway. Each pair of students selects 2–3 strains from each of these experiments for sequencing analysis based on visual assessment of beta-carotene production, resulting in a total of approximately 15–20 strains analyzed per semester. While the throughput of this genetic screen could be increased, allowing each group to select and work with their own strains results in a sense of ownership, as groups compete to see which strain will produce the highest beta-carotene yield. Moreover, even with this simple, low-throughput genetic screen, student projects in this course have resulted in novel findings. For example, the deletion of the gene Mcr1, which encodes a mitochondrial NADH-cytochrome b5 reductase that is involved in ergosterol biosynthesis (Hahne et al., 1994; Lamb et al., 1999), results in increased production of beta-carotene (Figure 1B and M. Calzini and M. Whittaker, unpublished data). Discovering new phenotypes and identifying novel roles for genes in the production of beta-carotene encourages students to investigate the literature and to design future experiments to further enhance desired phenotypes. In this way, these experiments model an authentic scientific process and facilitate student agency (Hester et al., 2018).

Importantly, sufficient time is built into the lab schedule to allow groups to repeat experiments when necessary, rather than providing students with “back-up” PCR products or transformants. This iteration further promotes ownership, as students carry out every step of their experiment, and have the opportunity to learn from mistakes and master new technical skills.

## Lecture Component: Engagement With Social Issues

### Engagement With Social Issues: Case Studies

The lecture portion of the course focuses on applications and societal implications of yeast metabolic engineering to achieve the fifth element of CUREs: broader relevance (Auchincloss et al., 2014; Brownell and Kloser, 2015). Student engagement with these topics is achieved through the use of three case studies. Cases have been shown to provide realistic scenarios that require critical thinking within a structure that can be used to promote engagement, motivation, and information retention (Allchin, 2013). Cases provide *context* and can be used to connect real-world problems with the topics or technologies discussed in the course. More than teasers and “hooks,” cases can be scheduled in a course to bring forth societal issues that complement lab techniques. We sequenced the cases we developed to intentionally introduce current applications and societal challenges addressed by the use of engineered yeast (Table 1). Moreover, a common thread was the emphasis on the sustainability of these approaches and their impact on *all* members of our society.

**TABLE 1:** Summary of case studies used to engage students in discussion of ethical and societal issues raised and addressed by advances in yeast metabolic engineering.

Case study and associated publications	Yeast species used	Metabolic engineering approach	Impact on sustainability	Ethical questions and societal issues
Impossible Burger (PLoS, 2019; Wired, n.d.; FAQ, n.d a)	*Pichia pastoris*	Introduction of a single gene encoding soy leghemoglobin	Creation of a meat-free burger; reduced emissions and water use	Can the Impossible Burger actually be considered vegan? Is the goal to produce a burger for vegetarians, or to convince meat-eaters to eat less meat?
Semisynthetic Opioid Production in Yeast (Galanie et al., 2015; Saccharomyces Genome Database [SGD], n.d b)	*S. cerevisiae*	Introduction of 16 genes from five different organisms to encode an entire metabolic pathway	Reduced water and fertilizer use compared to production from poppies	Potential for individuals to create illicit drugs. Yield of desired product is very low – is this method feasible?
Engineered Yeast and Verlasso Salmon (Xie et al., 2015; FAQ, n.d b)	*Yarrowia lipolytica*	Introduction of multiple pathways genes using marker recycling to encode the metabolic pathway needed to produce omega-3 and omega-6 fatty acids into *Y. lipolytica*, which was used in place of feeder fish to feed salmon	Reduced need for feeder fish for farmed salmon	Engineered yeast released into the ocean. After initial investment and work, DuPont no longer uses this strategy - why?

Cases were assigned as digital Google Docs worksheets via Doctopus (Wright et al., 2014), and students were allowed to work in groups or independently. Each case was introduced in class, and students were given approximately 30–45 min to begin working. Groups continued collaborating through Google Docs to finish the case studies outside of class. All case studies included learning outcomes, background information (often videos and related articles), and a series of questions for analysis. Questions required students to think critically about how yeast was used to produce the Impossible Burger (Case Study 1), opioids (Case Study 2), and omega-3 fatty acids (Case Study 3). Several questions then asked participants to compare the methods and approaches used in the different case studies. All cases included questions that prompted participants to reflect and think about: (a) whether this is an example of yeast metabolic engineering, (b) whether the approach is “sustainable,” (c) why is yeast genetic engineering and/or metabolic engineering useful for the production of this substance, and (d) the ethical implications of the technology.

Groups received feedback in their Google Docs correcting any misconceptions and asking additional questions to push students to think more deeply about sustainability. Most students in this course have completed BIT 501: Ethical Issues in Biotechnology and have a basic understanding of bioethics and a framework for discussing the social implications of biotechnological innovations. Based on the previous coursework and academic background of our students, we expected students to focus largely on environmental sustainability in their group answers. During the class period following each case study, a class-level discussion was used to extend students’ understanding beyond environmental sustainability to include economic and social sustainability. Typically, one or two groups had already considered these facets of sustainability in their Google Docs, and their comments during the class discussion sparked other groups to expand their initial treatment of the sustainability implications of the technologies discussed. Following the class discussion, instructors summarized key points in a course announcement. Expected answers to the questions posed in the Google Docs case studies as well as examples of topics typically addressed during class discussions (including all aspects of sustainability, as well as ethical and social justice considerations) are included in the Case Study Keys.

### Engagement With Social Issues: Graduate Sustainability Project

In addition to case study-based learning, graduate students enrolled in the course were required to complete a 3- to 5-page report discussing the use of yeast metabolic engineering for sustainable production of food, resources, biofuels, or bioremediation, referring to at least three primary literature publications. In addition to explaining the techniques used to engineer yeast, optimize yield, and generate the product, students were asked to explore state, local, and, if applicable, campus-wide initiatives related to their project and to propose a future direction or application that relates to the current economic growth and needs of our state and campus.

This assignment further promotes engagement by allowing choice. Students typically choose to analyze the sustainable generation of a product related to their own research or personal interests. The ability to connect the project to existing interests and societal concerns helps students to contextualize the science and reflect on its implications. The format of the project and guidelines offer structure and intentionally include the evaluation of scholarly research (primary references), summaries of complex genetic engineering processes for a lay audience, and connections to local and national societal issues. The assignment includes at least one figure or table, and students often produce graphical abstracts.

Grading is based on a modification of specifications grading, with clear requirements for A, B, and C work. An opportunity to earn bonus points is included, and this is often met when students go beyond the stated elements of the project and include information about local/campus-wide initiatives, interviews, or research that is impactful. For example, one exemplary project discussed the production of pigments for solar panels using yeast metabolic engineering, an approach that is not yet developed commercially and is understudied but has tremendous potential. Another project included first-person interviews with the researchers working to develop a yeast metabolic engineering-based technology.

Importantly, topics addressed in this project often become starting points for discussions and assignments in future versions of the class. In this way, these student-driven projects form the basis for case studies used in subsequent semesters. For example, in Spring 2021, we will use a new case study comparing the production of pigments for solar panels to the production of pigments for textiles using yeast metabolic engineering. This new case study combines two previous graduate projects in a way that lets students explore the similarities and differences in methods, technical challenges, and societal implications. Although the evolution of graduate student projects into case studies for future iterations of the course necessarily occurs after the end of the semester, when students are no longer enrolled, students are invited to participate in case study development and offered co-authorship if the case study results in a publication.

## Discussion

This course provides an example of a course structure intentionally designed to increase ownership and agency through the incorporation of critical elements of CUREs while also engaging students through case studies and projects focused on connecting the science to societal issues. Participants have actively searched for local technologies, companies, and researchers using engineered yeast to continue discussions and propose future applications of yeast metabolic engineering. For the graduate sustainability project, students have interviewed researchers, found examples of these technologies being used on our campus, and connected these efforts to their own research.

As the class continues to evolve, we plan to work with students to extend discussions about yeast metabolic engineering and sustainability beyond the classroom through the creation of podcasts, a website, and other public-facing educational materials. We also plan to strengthen the connection between this course and the existing Ethical Issues in Biotechnology course by inviting instructors and students in that course to provide feedback to Yeast Metabolic Engineering students on their case studies and graduate projects. Furthermore, discoveries made in the context of this course will be used as starting points for future undergraduate independent research projects and course offerings. For example, libraries of mutants produced by the class can be further analyzed by students in a course focused on high-throughput technologies and automation (e.g., BIT 479/579 *High-throughput Discovery*). Future educational studies will assess the impact of these course structures and practices on learning gains, engagement in sustainability efforts in and out of class, and student agency.

The equipment needed to offer this course – shaking and stationary incubators, spectrophotometers, centrifuges, themocyclers, and gel electrophoresis equipment – is often accessible to teaching labs. Reagents are either commercially available (e.g., Zymo Research, TransOMIC) or can be obtained from academic labs. The full electronic lab manual for this course is available from the authors upon request.

Students are genuinely excited by the ability to engineer yeast and produce a commercially relevant product that can be readily connected to their lived experience. The striking phenotype of yeast producing beta-carotene combined with the number of mutant libraries and genetic screens that can be adapted make this lab module an option for numerous different courses, student levels, and institutions. The VEGAS approach can be used to assemble other genetic pathways of interest that can be selected for and linked to societal, environmental, and public health needs. Importantly, the skills gained in the lab working with the model organism combined with ethical and social justice discussions directly linked to the applications of these technologies reinforce the professional development skills required of modern-day molecular biologists.

## Data Availability Statement

The original contributions presented in the study are included in the article/Supplementary Material, further inquiries can be directed to the corresponding author.

## Author Contributions

CLG and CCG designed the course, adapted the lab experiments for teaching labs and course learning objectives, designed the case study and graduate project assignments, and wrote the manuscript. Both authors contributed to the article and approved the submitted version.

## Conflict of Interest

The authors declare that the research was conducted in the absence of any commercial or financial relationships that could be construed as a potential conflict of interest.
