# Supplementary material for: Using Metabolic Engineering to Connect Molecular Biology Techniques to Societal Challenges
Source: Front Microbiol. 2020 Nov 16;11:577004. doi: 10.3389/fmicb.2020.577004 (PMC7701299; doi:10.3389/fmicb.2020.577004)
Supplement: Supplementary file 1 [file Data_Sheet_1.docx]

# Supplementary Material

Case Study 1: The Impossible Burger Key

Case Study 2: Semisynthetic Opioid Production in Yeast Key

Case Study 3: Engineered Yeast and Verlasso Key

Introducing Case Studies

Graduate Sustainability Project Guidelines

Case Study #1: The Impossible Burger

# Learning Outcomes

- **Explain** to a general audience how the Impossible Burger was generated by engineering yeast.
- **Describe** potential future applications of this technology.
- **Discuss** whether this technology is “sustainable”.

# Background

*
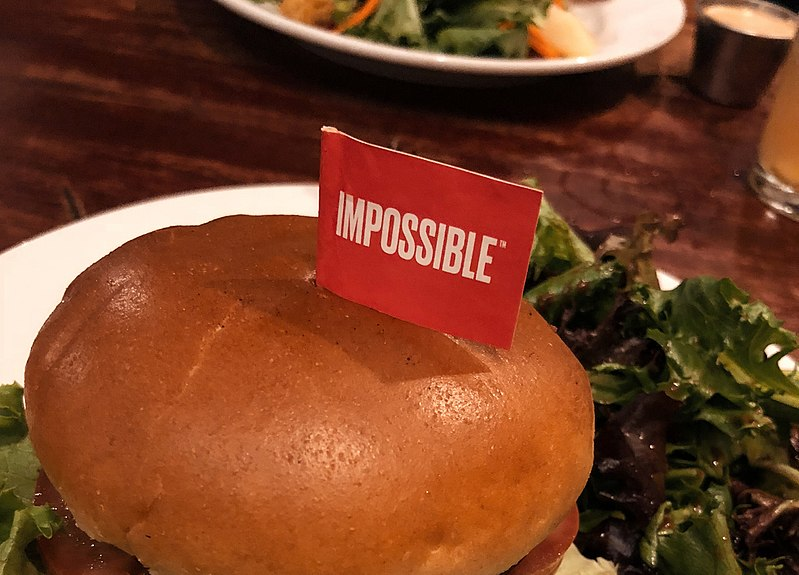
*

***The Impossible Burger.*** *Impossible Burger at Hell's Kitchen, Minneapolis - Vegan Meat.
Credit: Tony Weber, Creative Commons.*

*The Impossible Burger* has been in the news, and you may have enjoyed one of these plant-based burgers. *How is it made? Why is it innovative? How is it sustainable?*

Let’s start by watching this video [2:24 min].

<https://youtu.be/n6U4H8WC9jg>

Now read these articles:

<https://www.wired.com/story/the-impossible-burger/>

<https://blogs.plos.org/dnascience/2019/05/16/anatomy-of-an-impossible-burger/>

# Group Members

List the names of all the group members that participated in your group.


# Analysis

Working in small groups, respond to the following questions:

***Expected student answers in blue; additional topics for class discussion in red.***

1. Which component(s) of the Impossible Burger are produced in yeast?

Yeast are engineered to produce soy leghemoglobin. In addition, 40 components that are normally produced by yeast are included in the final burger.

Students discuss components of the burger made in yeast as well as those not made in yeast. They often discuss whether or not the burger can be considered vegan and to whom the burger is being marketed – is it really intended to be consumed by vegans and vegetarians, or is the goal to market the burger to meat eaters in an effort to decrease the total amount of meat consumed? A consideration often mentioned here is that the burger is commonly topped with conventionally produced cheese and other non-vegan products.

1. What yeast species is used? Why is this yeast useful for genetic engineering and/or metabolic engineering?

The Impossible Burger is produced using *Pichia pastoris*. *P. pastoris* has the following useful characteristics for genetic and metabolic engineering:

- It is a single-celled eukaryote.
- It is generally regarded as safe.
- It has a short doubling time and requires inexpensive media.
- You can also grow it with methanol to induce gene expression and function as a carbon source.

During the class discussion, we remind students of the definition of GRAS, a term previously introduced in lecture. While most students are comfortable with terminology related to research lab biosafety, they are often less familiar with terms used in industry.

In discussing the medium used for growth of *P. pastoris*, students may raise questions about the components of this medium, whether any of them are animal-derived, and whether this would impact the status of the burger as vegan or not vegan. In addition, students discuss the cost of the medium (economic sustainability) and the water consumption and electricity necessary to produce soy leghemoglobin in yeast.

Additional discussion of the technical aspects of soy leghemoglobin is often necessary. In particular, some students require additional background related to inducible gene expression and why the ability to induce the expression of a gene might be beneficial. With this first case study, students sometimes have the misconception that the goal is always to produce the largest quantity of the desired product possible.

1. Which gene(s) were introduced into yeast to create the Impossible Burger?

The *legHB* gene was introduced to create the Impossible Burger.

1. Is this an example of yeast metabolic engineering? Why or why not?

While we generally think of metabolic engineering in terms of introducing multiple genes encoding an entire pathway in order to produce a metabolite of interest, this is an example of the desired product being a single protein.

1. Refer to this list of ingredients: [Impossible Burger ingredients](https://faq.impossiblefoods.com/hc/en-us/articles/360018937494-What-are-the-ingredients-). Can you find any examples using **metabolically** engineered yeast to produce any of these ingredients? For one ingredient, list the following information:
   1. Ingredient:

Multiple answers are possible:

1. Vitamin B3. Technically they made nicotinamide riboside in our paper vs. niacin in the Impossible Burger, but both are B3 vitamins and are made along similar pathways. As a result, niacin can also be made using very similar methods.
2. Natural flavors
3. Sodium Ascorbate (Vitamin C)
   1. Link to paper using a yeast metabolic engineering approach to produce the ingredient:

Responses will vary. Examples are provided below.

1. Belenky P, Stebbins R, Bogan KL, Evans CR, Brenner C (2011). Nrt1 and Tna1-Independent Export of NAD+ Precursor Vitamins Promotes NAD+ Homeostasis and Allows Engineering of Vitamin Production. *PLoS ONE* 6(5): e19710. DOI: <https://doi.org/10.1371/journal.pone.0019710>
2. Denby, C.M., Li, R.A., Vu, V.T. *et al.* (2018). Industrial brewing yeast engineered for the production of primary flavor determinants in hopped beer. *Nat Commun* 9, 965 (2018). DOI: <https://doi.org/10.1038/s41467-018-03293-x>
3. Sauer M, Branduardi P, Valli M, Porro D. (2004). Production of L-ascorbic acid by metabolically engineered *Saccharomyces* *cerevisiae* and *Zygosaccharomyces* *bailii*. *Appl Environ Microbiol*. 2004;70(10):6086-6091. DOI: <https://doi.org/10.1128/aem.70.10.6086-6091.2004>
   1. Type of yeast used:
4. *S. cerevisiae*
5. Brewer’s yeast
6. *S. cerevisiae*
   1. Number of genes introduced to produce the ingredient:
7. *NRT1*, a nicotinamide riboside transporter, was deleted. So 0 genes introduced.
8. 5 genes: (*Ade2, tHMGR, FPPS*, McLIS, OtGES)*
9. One gene - ALO1. An additional gene, LDGH, can be added to increase production.
   1. Could this ingredient be produced in the same yeast currently used in Impossible Burger production? Why or why not? If so, would any additional genes need to be transferred?

Responses will vary, but students should consider whether necessary pathways and transporters exist in *P. pastoris*.

Discussion of these questions focuses on students sharing their findings and discussing aspects they found challenging to understand.

1. Is the Impossible Burger “sustainable”? Why or why not?

Responses will vary, but students should consider factors such as land and water use, emissions, and transportation requirements for production of Impossible Burgers versus traditional beef-based burgers.

Discussion typically focuses initially on environmental sustainability, with students thinking about the amount of land and water needed to raise cattle as well as the carbon emissions from the cattle themselves and transportation of those cattle. They compare this to the amount of water, electricity, and plastics they expect would be necessary to produce the Impossible Burger. They typically agree that they think that with appropriate scaling, the Impossible Burger would be less resource-intensive than a traditional burger.

Discussion of economic sustainability focuses on the cost of producing the Impossible Burger vs. a traditional burger as well as cost to consumer. Students typically raise questions about whether we can clearly say what the cost of production or the cost to consumer of either burger truly is, as the costs are related to government subsidies of agriculture and other federal, state, and local policies.

This discussion often leads directly to a discussion of how the cost of both burger types might differ from country to country, as different countries have different economic and environmental policies related to agriculture that will affect the price of beef – does a traditional hamburger cost about the same around the world, or not?

As students consider country-to-country differences in the economic sustainability of the Impossible Burger, they also often bring up different rules and regulations regarding the production and sale of genetically modified organisms in different countries – is the Impossible Burger even legal in all countries?

Entwined with discussions of to whom this product is targeted and what drives the cost of the product, students often bring up questions about who can afford to buy the Impossible Burger. Students tend to agree that the goal seems to be to encourage meat-eating individuals to eat slightly less meat, rather than to eliminate the production of traditional burgers entirely or to replace existing vegan or vegetarian products. Further, they tend to agree that this product is marketed to individuals with disposable income. This may lead to discussions of social implications of inequalities in access to food.

Throughout the discussion, students may question the ethics of producing and eating meat, and may discuss whether decreasing meat consumption should be a societal goal for environmental, economic, or ethical reasons, or a combination of all three.

Case Study #2: Semisynthetic Opioid Production in Yeast

# Learning Outcomes

- **Explain** to a general audience how scientists engineered yeast to make opioids.
- **Describe** potential future applications of this technology.
- **Discuss** whether this technology is “sustainable”.
- **Compare and contrast** the methods used in this study to those used to create the Impossible Burger.
- **Identify** and **explain** potential ethical implications.

# Background

*
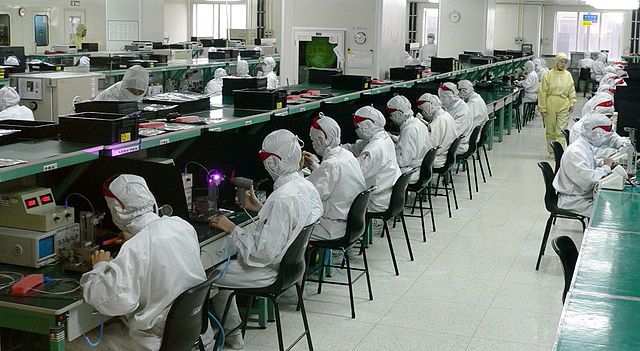
*

***Opioids****. “To make a tricky product you need to have the right factory, workers and machinery. And if you’re making opiate drugs, then yeast makes a great factory!” Image and quote from Wikimedia Commons and post on* [*https://www.yeastgenome.org/blog/tag/opiate-biosynthesis*](https://www.yeastgenome.org/blog/tag/opiate-biosynthesis)

*The Opioid Epidemic* has been in the news. *How are opioids made? Why is the use of yeast to make opioids innovative? How is it sustainable? What are the ethical and legal implications?*

Let’s start by reading two short blog posts:

<https://www.yeastgenome.org/blog/tag/opiate-biosynthesis>

Now read this abstract:

Opioids are the primary drugs used in Western medicine for pain management and palliative care. Farming of opium poppies remains the sole source of these essential medicines despite diverse market demands and uncertainty in crop yields due to weather, climate change, and pests. Here, we engineered yeast to produce the selected opioid compounds thebaine and hydrocodone starting from sugar. All work was conducted in a laboratory that is permitted and secured for work with controlled substances. We combined enzyme discovery, enzyme engineering, and pathway and strain optimization to realize full opiate biosynthesis in yeast. The resulting opioid biosynthesis strains required expression of 21 (thebaine) and 23 (hydrocodone) enzyme activities from plants, mammals, bacteria, and yeast itself. This is a proof-of-principle, and major hurdles remain before optimization and scale up could be achieved. Open discussions of options for governing this technology are also needed in order to responsibly realize alternative supplies for these medically relevant compounds.

[Galanie S, Thodey K, Trenchard IJ, Filsinger Interrante M, **Smolke** CD. Complete biosynthesis of opioids in yeast. *Science*. 2015 Sep 4;349(6252):1095-100. DOI: 10.1126/science.aac9373. Epub 2015 Aug 13. PMID: 26272907; PMCID: PMC4924617.](https://science.sciencemag.org/content/349/6252/1095.long)

# Group Members

List the names of all the group members that participated in your group.


# Analysis

Working in small groups, respond to the following questions. You may refer to the Galanie et al. paper referenced above or other information found online as necessary.:

***Expected student answers in blue; additional topics for class discussion in red.***

1. Which opioids can currently be produced in yeast?

Hydrocodone, morphine, codeine, oxycodone, hydromorphone and thebaine.

Students may discuss the medical and illicit uses of these drugs.

1. What yeast species is used? Why is this yeast useful for genetic engineering and/or metabolic engineering?

The authors use *S. cerevisiae*  to produce opioids. *S. cerevisiae* has the following useful characteristics for genetic and metabolic engineering:

- It is a single-celled eukaryote.
- It is generally regarded as safe.
- It has a short doubling time and requires inexpensive media.
- It is highly genetically tractable.
- Its genome is very well characterized.

As with the previous case study, students may question or discuss the composition of the yeast medium and the resources required for cultivation of the yeast and production of the opioids.

1. Which gene(s) were introduced into yeast to produce opioids? From what organism(s) were these genes derived?

*Rattus norvegicus* - SepR, PTPS, QDHPR, PCD, TyrH^WR^, BH_4_

*Pseudomonas putida:* DoDC, morB

*Coptis japonica*: NCS

*P. somniferum*: 6OMT, CNMT, 4’OMT, CPR, T6ODM

*Eschscholzia californica*: NMCH

Also, chimeric protein b/w P450 and SalSyn

1. Is this an example of yeast metabolic engineering? Why or why not?

Yes. The yeast are engineered to produce a desired metabolite by introducing multiple genes from multiple organisms encoding enzymes required to sequentially convert a substrate to the desired product.

1. Refer to the Galanie et al. paper. In addition to the opioids the authors were attempting to produce, what undesired intermediate accumulated at much higher levels than the desired products?

Neomorphine and reticulin accumulated at higher levels than the desired products.

1. Can you think of a way to drive the yeast to produce the desired products rather than this intermediate?

Responses may vary, as a strong chemistry background is necessary for students to arrive at the correct answer. The goal of this question is for students to think about this question before group discussion.

At the time of publication of the paper referenced in this case study, the isomerization of neopinone to codeinone was thought to occur spontaneously. A more recent publication (Dastmalchi *et al.* 2019) demonstrates that this process is actually catalyzed by neopinone isomerase, and yield of the desired products can be improved by introducing the gene encoding this enzyme into engineered yeast.

Dastmalchi, M., Chen, X., Hagel, J.M. *et al.* (2019). Neopinone isomerase is involved in codeine and morphine biosynthesis in opium poppy. *Nat Chem Biol* 15, 384–390. DOI: <https://doi.org/10.1038/s41589-019-0247-0>

Most student groups do not arrive at this answer in their GoogleDoc; it is typically a graduate student or chemistry undergraduate who is able to propose a way to drive this reaction. If no one has a suggestion at the time of discussion, students are given time to search for more recent papers to see if they can find the answer. We then pull up the paper as well as the lab website for this group and talk through this process as a group.

1. Is this a sustainable technology? Why or why not?

Responses will vary, but students should consider factors such as land and water use, emissions, and transportation requirements for production of opioids in yeast versus from poppies. In addition, students should consider the yield achieved for yeast-derived opioids.

Students typically consider the land and water use required for poppy cultivation vs. the resources needed to produce opioids in yeast. Most students come to agree that while production of opioids in yeast could theoretically improve upon the environmental sustainability of poppy-based production, the current method has an extremely low yield, and is thus quite resource-intensive.

They usually propose that in addition to not improving upon environmental sustainability, the yeast-based method probably does not improve upon the economic sustainability of producing opioids from poppies.

1. What are the ethical and legal implications of opioid production in yeast?

Responses will vary, but students should consider current regulations, illegal use and production of regulated drugs, the opioid epidemic, and purity and dosing.

Students are usually very familiar with the opioid crisis in parts of the United States, and generally discuss this question in the context of concerns about individuals using these methods to produce opioids from yeast in their homes. This suggestion tends to quickly return to the low yield of the current yeast-based process, and students agree that it would not be feasible to scale up enough to produce a significant amount of drug in a home-based lab.

In addition to wondering about the ability of individuals to produce illicit drugs at home, students often wonder about the typical cost of these drugs when prescribed for medical use and whether this process would decrease prescription drug price for patients. Students are allowed to perform quick internet searches to determine typical costs of these drugs both in the United States and in other countries. They are also encouraged to look into policies regulating the use of these drugs in various countries.

Throughout this conversation, students may also discuss the criminalization of drugs, how a government decides which drugs are legal, illegal, or legal but highly regulated, and how these decisions disparately impact individuals from different groups or communities.

Case Study #3: Engineered Yeast and Verlasso Salmon

# Learning Outcomes

- **Explain** to a general audience how scientists at DuPont engineered yeast and Verlasso used it for salmon production.
- **Describe** potential future applications of this technology.
- **Discuss** whether this technology is “sustainable”.
- **Compare** and **contrast** the methods used in this study to those used to create the opioid-producing yeast.
- **Identify** and **explain** potential ethical implications.

# Background

Let’s all watch this short video about the Verlasso salmon company:
 <https://youtu.be/xin-iKxaFtg> [2 min]

***Expected student answers in blue; additional topics for class discussion in red.***

What is the **problem**?

Commercial salmon fishing is not sustainable. Aquaculture approaches have been introduced to provide a more sustainable source of salmon, but aquaculture-based production requires a large number of feeder fish to achieve levels of omega-3 fatty acids similar to those found in wild salmon.

What **background** information do we have?

DuPont has engineered a yeast strain to produce omega-3 fatty acids which can be used as an alternative to feeder fish to more sustainably produce salmon in aquaculture systems.

This case study is administered slightly differently from the other two, in that on the day the case study is assigned, we watch the 2 minute video as a class, and then answer and discuss these first two questions as a class. The rest of the case study is completed in groups and discussed during the subsequent class session.

#

#

#

#

#

# Group Members

List the names of all the group members that participated in your group.


# Analysis

Working in small groups, respond to the following questions referring to the video (<https://youtu.be/xin-iKxaFtg>) and the article below.

Xie D, Jackson EN, Zhu Q. [Sustainable source of omega-3 eicosapentaenoic acid from metabolically engineered *Yarrowia lipolytica*: from fundamental research to commercial production. *Appl Microbiol Biotechnol*](https://www.ncbi.nlm.nih.gov/pmc/articles/PMC4322222/)*.* 2015 Feb;99(4):1599-610. DOI: 10.1007/s00253-014-6318-y. Epub 2015 Jan 8. PMID: 25567511; PMCID: PMC4322222. URL: <https://www.ncbi.nlm.nih.gov/pmc/articles/PMC4322222/>

##

## PART I: Background and Problem

#### The Study

Let’s now analyze the article entitled: [Sustainable source of omega-3 eicosapentaenoic acid from metabolically engineered *Yarrowia lipolytica*: from fundamental research to commercial production.](https://www.ncbi.nlm.nih.gov/pmc/articles/PMC4322222/)

What is ***Yarrowia lipolytica***?

*Y. lipolytica* is an aerobic yeast species that is able to grow in hydrophobic environments. This property makes it useful in the bioremediation of oil spills and production of lipid metabolites.

Students discuss how one might use *Y. lipolytica* to remediate oil spills – is it as simple as dumping the yeast into the ocean?

**Why** did the authors use this organism to produce omega-3 fatty acids?

*Y. lipolytica* is generally regarded as safe (GRAS) and is ideally suited for production and accumulation of lipids.

Why do you think **DuPont** was interested in this technology?

DuPont was likely interested in this technology because of its potential profitability as well as impact on DuPont’s image as an environmentally conscious company.

This part of the discussion focuses on the motives of large corporations. Do any corporations make choices solely because they believe it is the right thing to do, or because they want to have the image of being a company that makes socially responsible choices? Does it always matter if the product is profitable, or would a company take on a potentially profit-losing product in order to improve their image, and potentially increase sales of other, more profitable products? Does the intent of the company matter if the net impact contributes to environmental or economic sustainability and/or social justice?

### PART 2: The Approach

Summarize the key point of each figure in 1-3 sentences. Do not worry about experimental details -- focus on the take-away message.

**Figure 1.**

This figure shows the workflow used to genetically engineer *Y. lipolytica* to produce eicosapentaenoic acid (EPA) from sugar.

**Figure 2.**

This figure illustrates the metabolic pathway used by *Y. lipolytica* to produce linoleic acid and the additional engineered pathway to produce EPA. There are three possible pathways to produce EPA from linoleic acid, including the anaerobic polyketide synthase, aerobic desaturase, and elongase pathways.

**Figure 3.**

This figure illustrates the modular design of foreign genes introduced into *Y. lipolytica*. Each gene consisted of a promoter region, coding sequence, and terminator. This is analogous to the Transcriptional Unit system used in Yeast Golden Gate.

**Figure 4.**

This figure outlines the auxotrophic marker recycling strategy used to integrate multiple copies of foreign genes through homologous recombination.

**Figure 5.**

This figure illustrates the workflow used to generate new strains, screen them for production of omega-3 fatty acids, and scale up for commercial-scale fermentation.

**Figure 6.**

This figure demonstrates that similar biomass of EPA was produced via pilot scale, lab scale, and microscale fermentation.

**Figure 7.**

This figure depicts the production stages of EPA in lab-scale fermenters. The nitrogen source used during the growth phase is NH_4_OH to control pH as biomass is built up. The cultures are then shifted to nitrogen-limited conditions during the production phase in order to activate starvation-induced promoters and maximize EPA production.

**Figure 8.**

This figure shows the results of a simulation used to predict EPA production under different conditions.

Now, taking the paper as a whole, summarize the **approach** and **results** in 3-5 sentences.

The researchers first introduced multiple copies of multiple codon-optimized genes encoding metabolic pathways to produce EPA in *Y. lipolytica* using a marker recycling strategy. They performed a high-throughput screen of engineered strains under fermentation conditions and selected strains for optimization under fermentation conditions and scale up using a micro-fermenter system. Following strain selection, the authors performed additional medium and process optimization for each selected strain. They developed a two-phase batch fed system in which the growth phase is used to increase *Y. lipolytica* biomass, and the oleaginous phase is used to accumulate the desired lipids and used computational modeling to predict the response of selected strains to changes in conditions. Finally, they used these data to scale production up.

### PART 3: The Results and Implications

#### Sustainability.

Is this a **sustainable source of omega-3 fatty acids**? Why or why not?

Responses will vary, but students should consider overfishing, need for feeder fish, and resources needed for the production of omega-3 fatty acids in yeast.

Students often refer back to the video in their discussion. The problem was laid out for them there: overfishing of salmon presents environmental and economic challenges. Students may also discuss the disparate impacts of overfishing on different societies and cultures. As they discuss farmed salmon, they discuss the requirement for feeder fish, and how to make the use of feeder fish more sustainable. The video proposed that using omega-3-producing yeasts in feed could reduce the need for fatty acid-rich feeder fish, suggesting that Verlasso should result in increased environmental and economic sustainability for farmed salmon.

How does this product **affect** aquaculture, omega-3 fatty acids production, and the environment?

Responses will vary. Students should consider positive environmental impacts through enhanced sustainability of salmon aquaculture as well as potential environmental impacts of unintended dispersal of engineered yeast in the ocean.

The main topic of discussion related to this question is unintended dispersal of the Verlasso yeast, and the students typically conclude that this method has in fact resulted in the release of genetically modified yeast into the ocean. This brings them back to their previous discussions of differences in the regulation of GMOs in different countries, with a new twist: who controls the use of GMOs in the ocean?

How do you think **society** will respond?

Responses will vary.

#### Ethical implications

What are the **ethical** implications of this technology?

Responses will vary. Students should consider the ethical implications of the intended introduction of genetically modified yeast into the environment.

For both this question and the previous question, students generally weigh the need for increased sustainability in fisheries with public concern about GMOs.

What are **your thoughts** about this and related technologies?

Responses will vary.

Now read page 3 of this Executive Summary:

<https://www.seafoodwatch.org/-/m/sfw/pdf/reports/s/mba_seafoodwatch_verlassofarmedsalmonreport.pdf>

… and the FAQs found on Verlasso’s website: <https://www.verlasso.com/faq>

Does this **change** your thoughts about the engineered yeast? **Why or why not?**

Responses will vary. The linked documents should raise doubt about the environmental impact and sustainability of the engineered yeast and the Verlasso salmon.

Students tend to come out of these articles skeptical about the utility of omega 3-producing yeast for this application. They have concerns about the actual impact of this method on environmental and economic sustainability along with concerns about the ethics of releasing GMOs into the ocean.

#### Future applications

The papers and video you’ve considered are now a few years old, but the documents from SeafoodWatch and Verlasso are more recent. Now that you know how the technology developed and how it has been used commercially, can you find any updates or improvements to the technology itself? Provide a reference for a newer paper and a 3-5 sentence summary below.

Responses will vary. Examples of papers and summaries are included below.

1. Ji X-J, Ren L-J, and Huang H (2015) Omega-3 biotechnology: a green and sustainable process for omega-3 fatty acids production. *Front. Bioeng. Biotechnol.* 3:158. DOI: <https://doi.org/10.3389/fbioe.2015.00158>

This literature review discusses three major topics related to the production and usage of omega-3 fatty acids. In addition to discussing the synthesis of EPA synthesis in *Y. lipolytica*, the authors discuss DHA production in algal species and downstream uses of omega-3 fatty acids in oil extraction and refinement. While these technologies have not been studied to an extent to which they are ready to be applied commercially, they pose viable options for sustainable production of omega-3 fatty acids.

1. Xie D, Miller E, Sharpe P, Jackson E, and Zhu Q. (2017). Omega‐3 production by fermentation of *Yarrowia lipolytica*: From fed‐batch to continuous. *Biotechnol. Bioeng.*, 114: 798-812. DOI: <https://doi.org/10.1002/bit.26216>

Researchers took the fermentation process and methodology used in the paper from the case study and optimized it to produce more EPA. A two-stage fermentation process was shown to yield the most EPA in *Y. lipolytica*. The two-stage process was shown to increase productivity by up to 80%. The authors also stated that this method is not only suitable for large scale omega-3 fatty acid production, but also improves production in non-growth or partial-growth conditions.

1. Ferreira R, Gonçalves Teixeira P, Siewers V, Nielsen J. (2018). Free fatty acids from phospholipids in yeast. *Proceedings of the National Academy of Sciences* Feb 2018, 115 (6) 1262-1267; DOI: <https://doi.org/10.1073/pnas.1715282115>

The researchers altered *S. cerevisiae* to make them unable to utilize or store free fatty acids. This caused the yeast to export the fatty acids. The authors’ findings support the use of this method to develop yeast as a possible source of hydrocarbons for biofuel production.

Do you think the fatty acids produced using the technology described in the paper you found will be used in the same way as the fatty acids produced by DuPont? If not, can you think of other potential uses?

Responses will vary based on the paper selected by the group.

# References

1. Xue Z, Sharpe P, Hong S. *et al.* Production of omega-3 eicosapentaenoic acid by metabolic engineering of *Yarrowia lipolytica*. *Nat Biotechnol* 31, 734–740 (2013). DOI: <https://doi.org/10.1038/nbt.2622>
2. Xie D, Jackson EN, Zhu Q. Sustainable source of omega-3 eicosapentaenoic acid from metabolically engineered *Yarrowia lipolytica*: from fundamental research to commercial production. *Appl Microbiol Biotechnol*. 2015;99(4):1599-1610. DOI: <https://doi.org/10.1007/s00253-014-6318-y>
3. Zhu Q, Jackson EN. Metabolic engineering of *Yarrowia lipolytica* for industrial applications. *Curr Opin Biotechnol*. 2015;36:65-72. DOI: <https://doi.org/10.1016/j.copbio.2015.08.010>

Introducing Case Studies

The case study was distributed to students as a GoogleDoc using Doctopus prior to class. The GoogleDoc contained all needed background information, links to papers to be used in the case, and space to answer questions.

Students were instructed to work in small groups (3-4) of their choosing, with each group completing their case study together and submitting a single GoogleDoc. For each case, students were reminded that their goal was not just to dissect the techniques used to create the product of interest, but to wrestle with the societal implications.

Groups were given ~20-30 minutes to work together in class, and were then instructed to continue collaborating via GoogleDocs to complete the case study before the next class meeting.

During the following class meeting, groups discussed their answers.

Graduate Sustainability Project Guidelines

# Specific Student Learning Objectives:

1. **Summarize** applications of yeast metabolic engineering for sustainability-related projects such as renewable foods, chemicals, and fuel sources or remediation of the environment.
2. Critically **evaluate** the use of yeast metabolic engineering for sustainability-related projects.
3. **Connect** this research to current local, regional, state, and national needs and areas of economic and scientific growth.

# Assignment:

Write a three to five-page report discussing the use of yeast metabolic engineering for sustainable production of food, resources, biofuels, or bioremediation. This report should review the existing literature on the topic and connect it to state, local, and, if applicable, campus-wide initiatives.

Begin by reviewing the sustainability efforts of NCSU: <http://sustainability.ncsu.edu> to familiarize yourself with what sustainability entails. Next, search media outlets such as Futurity.org, the-scientist.com, scientificamerican.com, ScienceDaily.com, Science Friday, technologyreview.com, wired.com, New York Times Genetic Engineering column (found [here](http://topics.nytimes.com/top/news/science/topics/genetic_engineering/index.html)), and GenEngNews.com for news describing the engineering of yeast for sustainable products and processes. Then, dive deeper by searching PubMed.gov for the primary literature sources of these studies. Review at least three studies related to the topic you focus on and write a report that summarizes the goals of these studies, approaches used, and future directions. Finally, propose a future direction or application based on what you have researched that relates to the current economic growth and needs of our state and campus.

# Estimated Completion Time:

- Approximately four two-hour sessions (8 hours in total).

# Format:

- You may work in groups of two or three BIT 580 students.

# Milestones:

- Select topic and produce two related news articles by 1/24 (feedback provided).
  - We provide a [template](https://docs.google.com/document/d/1fFSF_WxvpFpPEPrIq2IdllOQ6DJKKUBLwCTHYCU1L-Q/edit?usp=sharing).
- Write an outline of the paper by 2/7 (feedback provided)
- Submit final paper (online) by 2/17 (feedback provided and final grade on assignment)

# Evaluation Method and Guidelines:

With no exceptions, reports will be evaluated based on the criteria listed below. Failure to include any one of the ten requirements will result in a drop in your grade and evaluation based on the criteria for the next lower grade. Within A work, for example, the inclusion of work beyond the requirements that strengthens the report by providing examples or applications or highlighting gaps in current knowledge may result in additional points awarded to achieve A+ (97-100) grade range, for example.

**A** work (**95**):

1. The document was submitted online (using Moodle) and before the deadline.
2. The document consists of 3-5 pages of single-spaced text (11 pt. font).
3. Report cites at least six references, three of which should be primary literature sources. In-text citations are properly referenced, and reference section is included.
4. In total, the document contains less than two typos, spelling errors, or grammatical mistakes.
5. The text is logically organized in clearly defined sections.
6. The report contains at least one table, infographic (see BioRender or Piktochart.com), flowchart (see LucidChart.com), or figure that summarizes the technology and its implications.
7. The report clearly reviews existing yeast metabolic engineering research related to the selected sustainability topic.
8. The report contains a text box with a 250- to 300-word lay audience summary.
9. Proposes future applications or directions for this technology by connecting it to regional and statewide needs and growth sectors.
10. The report ends with a clear but brief summary.

**B** work (**85**):

1. The document was **submitted online** (using Moodle) and before the deadline.
2. The document consists of **<3 or >5 pages of single-spaced text (11 pt. font).**
3. Report cites at least **four** references, two of which should be primary literature sources. In-text citations are properly referenced, and reference section is included.
4. In total, the document contains less than **three** typos, spelling errors, or grammatical mistakes.
5. The text is *logically* **organized** in clearly defined sections.
6. The report contains at least **one** table, infographic (see BioRender or Piktochart.com), flowchart (see LucidChart.com), or figure that summarizes the technology and its implications.
7. The report clearly reviews existing yeast metabolic engineering research related to the selected sustainability topic.
8. The report contains a text box with a **lay audience summary** that is longer than 300 words.
9. Proposes future applications or directions for this technology by **connecting** it to regional and statewide needs and growth sectors.
10. The report ends with a clear but **brief summary**.

**C** work and below (<**70**)

1. The document was **submitted online** (using Moodle) and before the deadline.
2. The document consists of **<3 or >5 pages of single-spaced text (11 pt. font).**
3. Report cites **two** references, one of which should be a primary literature source. In-text citations are properly referenced, and reference section is included.
4. In total, the document contains more than **four** typos, spelling errors, or grammatical mistakes.
5. The text is *logically* **organized** in clearly defined sections.
6. The report contains at least **one** table, infographic (see Piktochart.com or BioRender.com), flowchart (see LucidChart.com), or figure that summarizes the technology and its implications.
7. The report reviews existing yeast metabolic engineering research related to the selected sustainability topic.
8. The report contains a text box with a **lay audience summary** that is longer than 300 words and not clear.
9. **Does not propose future applications or directions** for this technology or **connects** research to regional and statewide needs and growth sectors.
10. The report **does not have a conclusion.**

An editable Word document [template](https://docs.google.com/document/d/1fFSF_WxvpFpPEPrIq2IdllOQ6DJKKUBLwCTHYCU1L-Q/edit?usp=sharing) is available.
